# Filariasis—III

**Published:** 1908-06-13

**Authors:** George C. Low

**Affiliations:** Lecturer in Tropical Diseases, West London Hospital.


### TROPICAL DISEASES.

### FILARIASIS.—III.

By GEORGE C. LOW, M.A., M.B., Lecturer in Tropical Diseases, West London Hospital.

The diseases that may be caused by the presence of *Filaria bancrofti* in the lymphatic system of man are numerous. It is only the adult forms that may excite pathological lesions; the embryos apparently are harmless. In many instances even the adults may live on and cause no appreciable trouble. Though this is very usually accepted, it is quite possible that if cases were closely followed for some years symptoms might ultimately develop. In countries where filariasis is common a special class of diseases affecting the lymphatic system will also be found to prevail. These comprise lymphangitis, dilated lymphatics, lymph scrotum, abscesses in connection with lymphatics, chyluria, chylocele (chyle in the tunica vaginalis), chylous ascites, chylous effusion into the pleural cavities, orchitis, and elephantiasis.

Before giving a brief description of some of these conditions it will be convenient first to discuss the pathology of their origin. There are a few observers who doubt the possibility of the filaria being a causative agent in their production at all. Granting, however, that it is difficult to explain the exact production of some of the lesions, there are yet many proofs of a mutual relation. The filaria in some way or other causes blockage and stenosis of the thoracic duct or other lymphatics; and this together with inflammation, generally due to cocci of some sort (streptococci, for example), gives rise to the lesions above mentioned. One of the simplest of the lesions caused by the filaria is dilatation of the superficial lymphatics of the arm or leg. Maitland, in India, by dissecting such nodosities has demonstrated the adults *in situ*, generally coiled up in little masses, and it is easy to understand that if in nature the parasites should die and become infected with organisms, an abscess might result. It was in such an abscess, as a matter of fact, that Bancroft, in whose honour the parasite is named, first discovered the adult forms. Less easy of explanation are the peculiar attacks of lymphangitis that filariated individuals are prone to. An individual, whether in his first or a subsequent attack, may suddenly get pain and tenderness in one limb; at the same time the temperature rises rapidly to 103° or 104° F., there may be a severe rigor, the leg gets red and swells, severe constitutional symptoms, vomiting, and delirium may be present, and the glands draining the implicated area enlarge and become very tender and painful. Without any treatment other than rest in bed all these symptoms subside in three or four days, and the swelling disappears. In the course of time the patient gets a similar attack, excited by such trivial causes as a scratch, a blow, or a sea bathe; and after several of these a careful examination of the limb attacked demonstrates that it is larger than its fellow. Each further exacerbation adds to this permanent thickening, and the early stage of elephantiasis is reached.

Case after case will give such a history, though in very rare instances there may be very little, if any, lymphangitis during the onset of this malady. Pathologically it would seem that both stasis of lymph and inflammatory attacks are preliminaries to that enormous hypertrophy of the tissues which rivals, without exaggeration, the size of the limbs of elephants. The legs, scrotum, penis, and vulva are the parts most usually affected; less commonly the arms, breast, ears, and scalp. Other lesions that may occur are dilated lymphatics in the groins (varicose groin glands), and these are important on account of their sometimes being mistaken for herniæ. They are soft and fluctuant, and when pressed on disappear, reappearing again when the pressure is taken off. If punctured with a hypodermic needle fluid like milk (chyle) can be drawn off, and in it young filarial embryos may be demonstrated. In other cases chyle may be passed with the urine. Young embryos may be demonstrated also sometimes in this fluid. The explanation of chyluria, chylocele, and chylous ascites is block or damage in the thoracic duct, producing a back flow into the superficial lymphatics. These channels become dilated, and if they rupture chyle is poured out into the bladder, tunica vaginalis, or peritoneal cavity, as the case may be.

The scrotum in some cases becomes enlarged (lymph scrotum). When the disease attacks the generative organs frequent attacks of lymphangitis take place from time to time; if these affect constituents of the spermatic cords they are very distressing to the patients. After severe attacks of lymphangitis it is very common for all the embryos to disappear from the blood. This simply means that their passage has been shut off by the inflammation, or that the adults have been killed. The same holds good in elephantiasis, which is a late sequel of the previous presence of the worms.

